# Explaining the U-Shaped Development of Intent-Based Moral Judgments

**DOI:** 10.3389/fpsyg.2016.00219

**Published:** 2016-02-18

**Authors:** Francesco Margoni, Luca Surian

**Affiliations:** Department of Psychology and Cognitive Sciences, University of TrentoRovereto, Italy

**Keywords:** moral judgment, moral development, intention, executive function, theory of mind

## Abstract

When preschoolers evaluate actions and agents, they typically neglect agents’ intentions and focus on action outcomes instead. By contrast, intentions count much more than outcomes for older children and adults. This phenomenon has traditionally been seen as evidence of a developmental change in children’s concept of what is morally good and bad. However, a growing number of studies shows that infants are able to reason about agents’ intentions and take them into account in their spontaneous socio-moral evaluations. Here we argue that this puzzling U-shaped trajectory in children’s judgments is best accounted for by a model that posits developmental continuity in moral competence and emphasizes the effect of immature executive function skills on preschoolers’ performance.

Mental state reasoning is required in several tasks, from inferential communication and the interpretation of social situations to the socio-moral evaluation of actions and agents. Children at an early age start to accuse peers by crying loudly “you did it on purpose!,” and legal systems typically distinguish harmful acts that are performed intentionally from acts that accidentally produce personal harm. A large body of developmental research investigated when and how children acquire the ability to attend to agents’ intentions and action outcomes in their socio-moral judgments, but the conclusions one can draw from infant studies seem at odds with the conclusions one can draw from studies on older children. Infants seem to possess abilities that young preschoolers’ responses do not reveal. In the present work, we address this puzzle by first reviewing relevant results on socio-moral reasoning in infants and children. Then, we evaluate different proposals put forward to explain the reported developmental changes and the apparent contradiction between infants and preschool children’s responses.

## The Outcome-To-Intent Shift in Preschoolers’ Moral Reasoning

Since Piaget’s (1932/1965) seminal work, a large body of studies has shown that a crucial developmental change from an outcome- to an intent-based moral evaluation occurs in the late preschool years. A typical Piagetian task would consist of evaluating which of two characters is more naughty and deserves to be punished. Piaget presented children with a story in which a supposedly well-intentioned character accidentally caused serious material damage (e.g., he broke 15 cups), and another story in which a bad-intentioned character caused, also by accident, less serious damage (e.g., he broke one cup). Younger children (aged 6–7) judged the character that produced serious material damage to be more naughty and punishable, whereas older children judged the bad-intentioned one to be more naughty and punishable. These and other similar findings were taken as evidence of a shift from an initial outcome-based (‘objective’) moral judgment to a later intent-based (‘subjective’) moral judgment.

Subsequent research overcame several methodological limitations of Piaget’s work (King, 1971; Farnill, 1974; Karniol, 1978; Nelson, 1980), but confirmed the occurrence of an outcome-to-intent shift. During the 1970s and 1980s, many different tasks were developed to investigate this phenomenon. By reducing the cognitive processing necessary to answer experimenters’ questions, scholars found that even preschoolers, at age 3, can attend to agents’ intentions in their moral evaluations (e.g., Armsby, 1971; Farnill, 1974; Yuill, 1984; Yuill and Perner, 1988). Nevertheless, Piaget’s main claim concerning the outcome-to-intent shift found further support, since children older than 4–5 years relied more on intention and less on outcome, whereas younger children showed the opposite pattern (e.g., Costanzo et al., 1973; Imamoglu, 1975; Keasey, 1978; Moran and O’Brien, 1983; Baird and Astington, 2004; Wainryb et al., 2005; Nobes et al., 2009; Cushman et al., 2013).

When intentions and outcomes lead to conflicting responses, as in the cases of *failed attempts to harm* and *accidental harm*, young preschoolers attend to outcome more than older children, relying mostly on outcome (e.g., Helwig et al., 1995), or equally on intention and outcomes (e.g., Killen et al., 2011; Cushman et al., 2013). With age, the condemnation of attempted but failed harm increases (Helwig et al., 1995), whereas the condemnation of accidental harm decreases (Cushman et al., 2013; see also Killen et al., 2011 on the development of an intent-based punishability evaluation).

While intentions dominate adults’ attribution of moral goodness and badness, adults often rely on both intent and outcomes to evaluate the punishability of agents (Cushman, 2008). A recent dual-process model explains why this is so: adults’ moral reasoning is generated by the work of two independent and sometimes conflicting processes, one that attributes value to actions and assesses agents’ mental states, and the other that evaluates the causal responsibility for action outcomes (Cushman, 2013, 2015). This proposal contradicts the Piagetian view, which posited a full replacement of the outcome-based judgment by an intent-based judgment.

## Infants’ Intent-Based Socio-Moral Evaluations

Extrapolating the developmental trajectory found in preschoolers, one may predict that infants and toddlers would rely mostly on action outcome rather than agents’ intention, assuming that they can produce a moral judgment. However, recent evidence shows that this is not the case. Several studies suggest that, in the first year, infants are able to distinguish between intentions and outcomes, they evaluate helping, harming and distributive actions, and they rely, for these evaluations, on intentions rather than outcomes.

Experimental studies used both elicited-response tasks and spontaneous-response tasks in the *violation-of-expectation paradigm*. Both research strategies found that, by the end of the first year, infants are able to attend to agents’ intentions and understand successful as well as failed actions (e.g., Woodward, 1998; Gergely and Csibra, 2003; Brandone and Wellman, 2009). This early understanding of failed attempts generalizes to first- as well as third-party socio-moral evaluations (Behne et al., 2005; Dunfield and Kuhlmeier, 2010; Hamlin, 2013; Lee et al., 2015).

In studies on *first-party* evaluations, infants were engaged in interactions with an experimenter and were presented with actors that were either unwilling or unable to please them (e.g., Behne et al., 2005; Dunfield and Kuhlmeier, 2010; Marsh et al., 2010). While the outcomes were identical in both conditions, intentions were different (negative for ‘unwilling agents,’ positive for ‘unable agents’). Infants responded differently to these two cases, showing that they used intention cues to guide their *first-party* evaluations and preferences. Nine-month-olds’ spontaneous signals of impatience (such as reaching and banging or looking and turning away) revealed that they become more agitated when they interact with actors unwilling to provide them with a toy (Behne et al., 2005). Moreover, using a manual choice measure (infants have to choose between two contrasted individuals), some studies found that by the second year of life, infants choose to help an unable over an unwilling actor, when asked to help someone. By contrast, infants were equally likely to help able agents, who displayed positive intentions and successful actions, and unable agents, who displayed positive intentions and unsuccessful actions (Dunfield and Kuhlmeier, 2010). Overall, these studies show that infants process information about intention and use it to evaluate others’ behavior.

Further studies on infants’ representations of harm and help examined *third-party* socio-moral evaluations (e.g., Kuhlmeier et al., 2003; Hamlin et al., 2007, 2013; Hamlin and Wynn, 2011; Meristo and Surian, 2014). Infants observed events in which an agent either helps or hinders the goal-directed action of another agent. Their evaluations of prosocial and antisocial actors were typically tested using a manual preference task. Early in their first year of life, infants consistently prefer the helper over the hinderer (Hamlin et al., 2007; Wynn, 2008). Also, at 16 months they prefer agents performing fair over unfair distributive actions (e.g., Geraci and Surian, 2011). When evaluating an agent’s behavior, infants are able to take into account not only a person’s intention, but also other relevant mental states such as informational states and beliefs (Hamlin et al., 2013; Meristo and Surian, 2013; Choi and Luo, 2015; for a review: Baillargeon et al., 2015).

Hamlin (2013; see also Hamlin et al., 2013) played a puppet show in which puppets either try but fail or succeed to help (or hinder) someone’s goal-directed action. Eight-month-olds preferred a helper (failed or successful) over a hinderer, but, most importantly here, infants did not prefer the successful helper (displaying both intention and relevant outcome) over the puppet that attempted to help, but failed (showing a good intention, but no relevant outcome). This suggests that infants’ preferences were guided by agents’ intentions rather than outcomes. Moreover, studying expectations by measuring spontaneous looking behavior, scholars recently found that by the end of the first year, infants infer agents’ socio-moral preferences by taking into account the agents’ information about others’ prosocial and antisocial intentions (Lee et al., 2015). They expect that an agent would prefer to approach a second agent who has previously shown a good intention, no matter what the consequences of the second agent’s action were.

How can we reconcile the classic results of preschoolers’ outcome-to-intent shift with these recent results of infants’ intent-based expectations and evaluations? A lesson may be learned from the literature on theory of mind.

## How to Account for Seemingly Conflicting Results: The Case of False Beliefs Tasks

The description of the intent-based judgment development sketched above, that is, an initial intent-based evaluation developing from an outcome-based evaluation that in turn shifts again toward an intent-based evaluation, resembles the ‘*puzzle about belief’* (Perner and Roessler, 2012), that is, the puzzle regarding the development of Theory of Mind. Using traditional elicited-response tasks to study children’s attributions of false beliefs, researchers initially concluded that the ability to attribute false beliefs does not emerge until about the fourth birthday (e.g., Wimmer and Perner, 1983; Baron-Cohen et al., 1985; Wellman et al., 2001). However, using *violation-of-expectation* and *anticipatory-looking* spontaneous-response tasks, researchers began to study also infants’ mentalizing abilities. Using these and others tasks, scholars demonstrated that babies at least in their second year of life are able to attribute reality congruent and incongruent mental representations across several situations (Southgate et al., 2007; Surian et al., 2007; Buttelmann et al., 2009; Baillargeon et al., 2010; Luo, 2011; Low and Perner, 2012; Surian and Geraci, 2012).

Why do 3-year-olds fail to attribute false beliefs when their abilities are tested on elicited-response tasks? There are two possible answers to this question. First, one may posit continuity during development and argue that preschoolers fail because they do not have the necessary executive function skills to pass an elicited-response task. Second, one may posit a conceptual change during development and argue that the representations and processes involved in resolving spontaneous-response tasks are fundamentally different from the ones involved in resolving elicited-response tasks.

Young preschoolers may succeed in representing the agent’s false belief, as infants do, but fail to select the right response and inhibit the wrong response when they are questioned via an elicited-response task (Leslie and Polizzi, 1998; Baillargeon et al., 2010). An *innate modular account* posits that from an early age babies are able to represent and use others’ mental states to understand social situations (Leslie et al., 2005; Surian et al., 2007; Kovács et al., 2010). What really develops is the set of cognitive abilities that children need to exploit their representational skills. At 3 years, executive function skills are not sufficiently developed to meet the processing demands of the elicited-response tasks (Thoermer et al., 2012). The continuity account is receiving growing experimental support, but it is still controversial. Many argue for a *conceptual shift account* according to which infants that pass a spontaneous-response task show a qualitatively different level of understanding compared to children who pass an elicited-response task (e.g., Wellman, 2014).

## Reconciling Results on Infants and Children at the Processing Level

As in the literature on false–belief understanding, we can draw a distinction between two main positions. First, an *emergence* view posits that during preschool years a conceptual change occurs in moral competence. The construction of a novel conceptual competence explains why school-aged children’s judgments differ from preschoolers. This view does not deny the role of executive function skills, as these are certainly involved in theory construction and revision processes (Cushman et al., 2013). Second, an *expression* view posits conceptual continuity during development and sees the role of executive function in a very different way. It claims that developmental differences result solely from changes in executive function, or theory of mind, that are external to the moral competence (Zelazo et al., 1996; Chandler et al., 2001; Killen et al., 2011). We argue that the studies on infants’ spontaneous socio-moral evaluations we briefly reviewed above favor the latter view and challenge the former, assuming that a “rich interpretation” (Aslin, 2000) of the infant studies is the correct one. Studies on infants’ evaluations suggest that infants can employ an intent-based concept of moral goodness and badness in their socio-moral evaluations. Therefore, the development of intent-based moral judgment is unlikely to derive from a conceptual change occurring in the preschool years.

If infants already possess an intent-based concept of moral badness and goodness, can executive limitations account for preschoolers’ outcome-based judgments? The expression view claims that young preschoolers fail at weighting intentions more than outcomes because of processing demands of the task. The additional processing demands of the elicited-response tasks compared to the spontaneous-response task used in the infant literature, lead kindergartners to produce outcome-based evaluations. With the acquisition of sufficient executive function (roughly at 4), children’s responses on elicited-response tasks can gradually match infants’ spontaneous ones, and become mostly intent-based. When judging an action or an agent in elicited-response tasks, for preschoolers it is difficult to suppress cues concerning action outcomes, while older children may have the sufficient executive function abilities to inhibit an outcome-based judgment and select an intent-based response (or *set shift* to an intent-based response, see Miyake et al., 2000; Diamond, 2013).

Highlighting how different tasks tap different forms of evaluation, and distinguishing between elicited and spontaneous responses may provide the key to solving the *puzzle about intent-based moral judgment*, and avoiding two conclusions that appear highly implausible (Hamlin, 2013). First, it would be implausible that an early tendency to privilege intentions over outcomes emerges during infancy only to be replaced during preschool years by the opposite tendency to privilege outcomes over intentions, or to weight these cues equally, and eventually be again replaced with a final tendency to privilege intentions. Second, it would be also very odd to posit that infants’ evaluation system is not related to the later evaluation system, so that we would have two intent-based moral evaluations mutually independent. Conversely, it is likely that if an evaluation system emerges, it will not be replaced later in the development by a new system that serves the exact same function. In order to test directly the expression account, future research should also investigate young preschoolers’ generation of intent-based moral evaluations in spontaneous-response tasks.

## Additional Factors that may Affect the Outcome-To-Intent Shift

In an expression view, several internal and external factors may promote the emergence of an intent-based elicited response. Among the internal factors, we can include the frontal lobe maturation underlying the acquisition of executive functions (Huttenlocher and Dabholkar, 1997; Benes, 2001; Miller and Cohen, 2001; Moriguchi and Hiraki, 2009, 2011; Moriguchi, 2014; but see also Knight and Stuss, 2002; Lepsien and Nobre, 2006). Among the plausible external factors, one could include interactions with adults and peers (e.g, Tomasello et al., 2005). Preschoolers start to be considered somewhat responsible for their actions by their parents, and parents correct their behaviors by pointing to actions outcomes (Piaget, 1932/1965) or negligence (Nobes et al., 2009). However, this may not be true for infants and older school-aged children. While infants’ actions outcomes are limited in their valence and severity, and parents do not deem their children fully responsible for what they cause, older children develop a more controlled behavior, and parents or peers now privilege a comprehensive evaluation of children’s intentions and outcomes.

Now, moving from an explanation concerning proximal causes (the processing level discussed in the previous section), to an explanation concerning distal causes (the evolutionary level), one promising perspective is offered by the *life-history theory*. Life-history theory is an approach in evolutionary biology that seeks to explain the timing of the organism’s ontogenesis by linking it to relevant evolutionary pressures (Kaplan and Gangestad, 2005). The emergence of a trait in the phenotype has both costs and benefits for the organism with regards to its reproductive fitness. The timing of such emergence would optimize the costs/benefits trade-off by on-setting a certain trait at a particular age, rather than earlier or later. Originally, this perspective was employed to explain the timing of morphological and physiological traits, such as sexual maturation, but recently it has been argued that it may also explain children’s delay in acting accordingly to fairness principles (Sheskin et al., 2014). In fact, while infants appear to evaluate others following an implicit understanding of fairness and harm, only some years later they consistently apply those moral principles during their social interactions (Siegal, 1982).

How can one apply life-history theory to the development of intent-based moral reasoning? Advocates of life-history theory may want to claim that the elicited intent-based moral reasoning emerges roughly at 5–6 years because at this time-point children increasingly engage in social interactions with peers. To understand and properly evaluate others’ intentions is fundamental in forming and maintaining such relationships. Attending to agents’ intentions, rather than actions outcomes, in the evaluations of agents, may become crucial just at the age in which children, in the evolutionary past, could not rely anymore on ‘free’ resources provided by parents and had to rely on their interactions with peers, avoiding potentially dangerous conflicts (Marlowe, 2005). Therefore, life-history theory may explain both the growing concern for fairness and the growing reliance on agents’ intention in preschoolers. The defendants of this position may then conclude that the human mind is wired with an innate ability to understand and evaluate others’ intentions, but it is only during the late preschool years that this ability is systematically recruited by children in a variety of social interactions.

## Conclusion

In sum, we have seen that young preschoolers’ outcome-based judgment is preceded by an early capacity to evaluate intentions that is revealed in spontaneous-response tasks. Drawing a parallel with the literature on the acquisition of mental state reasoning, we argued that the outcome-to-intent shift is best explained by an expression account that posits an early emerging infant socio-moral competence and explains preschoolers’ outcome-based judgments as due to immature domain-general executive function. Current evidence is more consistent with a view that assumes developmental continuity than with the opposite view based on conceptual changes.

## Author Contributions

All authors listed have made substantial, direct and intellectual contribution to the work, and approved it for publication.

## Conflict of Interest Statement

The authors declare that the research was conducted in the absence of any commercial or financial relationships that could be construed as a potential conflict of interest.
